# Nirmatrelvir/ritonavir use reduces risk for long COVID in patients with immunodeficiency

**DOI:** 10.70962/jhi.20250107

**Published:** 2025-11-04

**Authors:** Karen M. Gilbert, Mostafa Aglan, Aditi Jogdand, Neha V. Khairnar, Henry Ssemaganda, Mei-Sing Ong, Jocelyn R. Farmer

**Affiliations:** 1 Department of Population Medicine, Harvard Pilgrim Health Care Institute, Boston, MA, USA; 2 Department of Internal Medicine, Lahey Hospital and Medical Center, Burlington, MA, USA; 3 Division of Allergy and Immunology, Clinical Immunodeficiency Program of Beth Israel Lahey Health, Lahey Hospital and Medical Center, Burlington, MA, USA; 4 Comparative Effectiveness Research Institute (CERI), Lahey Hospital and Medical Center, Boston, MA, USA; 5 Computational Health Informatics Program, Boston Children’s Hospital, Boston, MA, USA

## Abstract

A retrospective study indicates that nirmatrelvir/ritonavir treatment during acute SARS-CoV-2 infection significantly reduces the risk of long COVID in patients with immunodeficiency. These findings may support early antiviral intervention to mitigate long-term sequelae in this vulnerable population, though prospective validation is required.

## Teaser

Our retrospective study highlights that acute use of nirmatrelvir/ritonavir significantly reduces the risk of post-acute sequelae of SARS-CoV-2 infection (long COVID) in immunodeficient patients, emphasizing the need for clinical trials that include this high-risk population.

Post-acute sequelae of severe acute respiratory syndrome coronavirus 2 (SARS-CoV-2) infection, commonly known as long COVID, refers to persistent or worsening symptoms following the acute phase of coronavirus disease 2019 (COVID-19), affecting 10–35% of US adults (1). Currently, there are no Food and Drug Administration–approved treatments for long COVID, though the antiviral drug nirmatrelvir/ritonavir is being investigated for its potential to reduce long-term COVID-19 effects. While nirmatrelvir/ritonavir has proven effective in decreasing the severity of acute COVID-19, its impact on long COVID remains uncertain (2). Several studies suggest that nirmatrelvir/ritonavir can mitigate post-COVID symptoms such as insomnia, dementia, anxiety, and depression and reduce the overall risk for long COVID. However, other research found no significant alleviation of long COVID symptoms with nirmatrelvir/ritonavir, demonstrating high variation by study design, setting, and patient population (3). Here, we conduct the first study to investigate the association of nirmatrelvir/ritonavir use with risk of long COVID among patients with immunodeficiency—a particularly vulnerable cohort who are at high risk for severe SARS-CoV-2 infection and long COVID (4).

This study was approved by the Lahey Hospital and Medical Center (LHMC) Institutional Review Board of Beth Israel Lahey Health. We conducted a retrospective review of electronic health records (EHRs) from March 15, 2015, to November 27, 2024, starting at the first available EHR extraction date to ensure the capture of all patients with immunodeficiency who may have been diagnosed prior to the onset of the COVID-19 pandemic. We used Comparative Effectiveness Research Institute resources to facilitate accurate data extraction from the EHR’s Clarity database, followed by secure transfer to a Research Electronic Data Capture database hosted at LHMC. The study population comprised patients diagnosed with SARS-CoV-2 (B34.2, B97.29, or U07.1), long COVID (B94.8 or U09.9), and immunodeficiency (inclusive of inborn errors of immunity [IEIs]: predominantly antibody deficiency [D80.x and D83.x], combined immunodeficiency [D81.x], immunodeficiency with major defects [D82.x], lymphocytopenia [D72.810], functional neutrophil defects [D71.x], complement deficiency [D84.1], and secondary/other immunodeficiency: D84.x, D89.9, and R76.8), identified by International Classification of Diseases, 10th Revision (ICD-10) codes. We further restricted the cohort to only patients diagnosed with COVID-19 after December 2021 (the date of nirmatrelvir/ritonavir emergency use authorization). All immunodeficiency patients with long COVID were additionally chart reviewed and validated as either IEI or secondary/other immunodeficiency by a board-certified allergist/immunologist, including cases due to secondary iatrogenic immunosuppression, lymphocyte clonality, and immunoglobulin wasting.

To estimate the causal effect of nirmatrelvir/ritonavir use on long COVID risk, we applied inverse probability weighting (IPW) to account for potential confounders influencing the likelihood of receiving nirmatrelvir/ritonavir. Accordingly, inverse probability weights were assigned based on patient self-reported demographics (gender, age, race, and ethnicity), total number of COVID-19 vaccination doses, COVID-19 severity (defined as requiring hospitalizations and intensive care unit admissions for COVID-19), preexisting chronic lung disease (including asthma, bronchiectasis, and interstitial lung—J47, J48, and J84), and IEI diagnostic severity (categorized into mild, moderate, severe, and unknown in accordance with the specific IEI diagnosis). Race, ethnicity, and gender were self-identified by patients on entry into the EHR and were then directly extracted. SARS-CoV-2 severity and IEI diagnostic severity were included in the model to adjust for their potential impact on the development of long COVID. Additionally, preexisting chronic lung disease was included as confounder since it is a common comorbidity among patients with immunodeficiency and a known risk factor for severe SARS-CoV-2. To limit the influence of extreme weights in the IPW analysis, truncation was applied by setting a lower and upper bound at the 1st and 99th percentiles of the weight distribution, respectively. Logistic regression was applied to assess factors associated with the likelihood of receiving nirmatrelvir/ritonavir. All analyses were performed using the R statistical software (version 4.4.0). IPW analyses were conducted using the R package *ipw* (version 1.2.1). All analyses were two-sided, with a P value of <0.05 considered indicative of statistical significance.

There were 2,740 patients with immunodeficiency and a documented SARS-CoV-2 infection during the study period. 597 patients (21.8%) had primary immunodeficiency, and 2,143 patients (78.2%) had secondary or other immunodeficiency. Among these patients, 73 (2.7%) were diagnosed with long COVID, compared with 1.9% in the overall EHR cohort, and 1,062 (38.8%) received nirmatrelvir/ritonavir following their COVID-19 diagnosis. The median time to nirmatrelvir/ritonavir initiation was 0 days (interquartile range: 0–0) from the date of COVID-19 diagnosis. Female patients (Odds Ratio [OR] = 1.27; Confidence Interval [CI] = [1.08, 1.51], P = 0.004), those with a severe immunodeficiency (OR = 2.21; [1.48, 3.33], P < 0.001), those who received ≥1 COVID-19 vaccination (OR = 3.11; [2.40, 4.08], P < 0.001), and those diagnosed with a preexisting chronic lung disease (OR = 1.26; [1.07, 1.49], P = 0.005) were more likely to receive nirmatrelvir/ritonavir.

After adjusting for potential confounders in the IPW model, nirmatrelvir/ritonavir use was significantly associated with a reduced risk for long COVID (OR = 0.43; [0.24, 0.72], P = 0.002). Standardized mean difference for all covariates between the treatment and non-treatment groups following adjustment were <0.1, indicating good covariate balance (Table 1). The association between long COVID and nirmatrelvir/ritonavir persisted in a subgroup analysis comprising only patients with IEI, excluding secondary/other immunodeficiency (*n* = 597; OR = 0.28; [0.07, 0.85], P = 0.041), as well as in another subgroup analysis including only patients who were vaccinated against COVID-19 (*n* = 2,340; OR = 0.54; [0.30, 0.93], P = 0.030). Finally, we performed a sensitivity analysis including patients diagnosed with COVID-19 both before and after nirmatrelvir/ritonavir received emergency use authorization (*n* = 3,784). The association between long COVID and nirmatrelvir/ritonavir remained consistent (OR = 0.42; [0.30, 0.57], P < 0.001). Notably, among individuals who received nirmatrelvir/ritonavir, female patients were more likely to be diagnosed with long COVID than male patients (OR = 3.03; 95% CI [1.18, 10.3], P = 0.040). Gender was not associated with the risk of long COVID in the overall cohort (OR = 1.27; [0.94, 1.74], P = 0.123).

**Table 1.** Baseline characteristics of study cohort before and after applying IPW

Covariate	Unweighted			IPW weighted		
	No nirmatrelvir/ritonavir (*n* = 1,678)	Nirmatrelvir/ritonavir (*n* = 1,062)	SMD	No nirmatrelvir/ritonavir (*n* = 2,744.4)	Nirmatrelvir/ritonavir (*n* = 2,716.8)	SMD
Age, mean (SD)	62.3 (18.1)	64.8 (14.2)	0.155	63.4 (17.5)	63.8 (14.6)	0.022
Gender: male (%)	594 (35.4)	319 (30.0)	0.114	918.6 (33.5)	906.6 (33.4)	0.002
Race: non-white (%)	1,516 (90.3)	972 (91.5)	0.041	2,491.1 (90.8)	2,470.7 (90.9)	0.006
Total number of COVID-19 vaccinations	3.12 (2.1)	4.2 (2.1)	0.498	3.53 (2.2)	3.56 (2.11)	0.011
Ethnicity: Hispanic (%)	1,585 (94.5)	1,008 (94.9)	0.020	2,598.6 (94.7)	2,575.6 (94.8)	0.005
COVID-19 hospitalization (%)	161 (9.6)	35 (3.3)	0.259	195.6 (7.1)	180.2 (6.6)	0.020
COVID-19 ICU admission (%)	35 (2.1)	3 (0.3)	0.167	37.8 (1.4)	19.9 (0.7)	0.063
Chronic lung disease (%)	512 (30.5)	379 (35.7)	0.110	891.7 (32.5)	899.4 (33.1)	0.013
Severity of immune deficiency (%)	​	​	0.179	​	​	0.009
High	42 (2.5)	57 (5.4)	​	98.6 (3.6)	96.6 (3.6)	​
Mild	161 (9.6)	122 (11.5)	​	281.2 (10.2)	272.7 (10.0)	​
Moderate	115 (6.9)	88 (8.3)	​	205.0 (7.5)	207.1 (7.6)	​
Secondary or unknown	1,360 (81.0)	795 (74.9)	​	2,159.5 (78.7)	2,140.4 (78.8)	​

ICU, intensive care unit; SDM, standardized mean difference.

Our findings highlight the potential broad benefit of nirmatrelvir/ritonavir in reducing the risk of long COVID among patients with both primary and secondary forms of immunodeficiency. Long COVID risk reduction with nirmatrelvir/ritonavir use persisted despite controlling for (1) patient demographics, (2) preexisting chronic lung disease, (3) COVID-19 vaccination status, (4) severity of the underlying immunodeficiency, and (5) severity of the acute COVID-19 infection. These data critically expand on our prior published work showing that patients with IEI are at a higher risk for long COVID, even after adjusting for preexisting chronic lung disease and initial COVID-19 severity (4). These data align with emerging pathomechanism theories in long COVID that suggest dysregulated adaptive immune responses, such as impaired memory T cell function, may drive long COVID risk (5). Despite their vulnerability, immunodeficient patients have been excluded from several key long COVID trials, limiting the applicability of trial results to this population who may have unique responses to treatments due to their compromised immune systems. Our results underscore the necessity for more inclusive clinical trials that encompass high-risk populations, including those with immunodeficiencies.

Our study showed that among nirmatrelvir/ritonavir-treated immunodeficiency patients, women had a higher risk for long COVID compared with men. Further studies are needed to investigate the underlying causes of this observed gender-based difference.

Our study is limited by incomplete health records and variations in medical practices, which could influence the classification of immunodeficiency and COVID-19 outcomes. The single-center design limits generalizability, and the use of ICD-10 codes might not fully capture clinical complexity or confirmatory testing. Additionally, small sample sizes in certain groups make it challenging to detect subtle differences in long COVID risk or the effectiveness of nirmatrelvir/ritonavir. Focusing on post-nirmatrelvir/ritonavir cases might introduce bias, and changing COVID-19 conditions could affect the findings. To address these limitations, we conducted multiple sensitivity analyses, including subset analyses of dates before and after the emergency use authorization for nirmatrelvir/ritonavir as well as analyses limited to patients with a clarified primary immunodeficiency diagnosis. A board-certified immunologist reviewed all cases of unspecified immunodeficiency (D84.x, D89.9, and R76.8), reclassifying most of these diagnoses as secondary immunodeficiency. Finally, although we have adjusted for known factors associated with long COVID in our analyses, other unaccounted factors could have influenced the results. Additional SARS-CoV-2 antiviral and monoclonal therapies have emerged in clinical practice. Remdesivir was not administered to any patients in this cohort. Analysis of other anti–SARS-CoV-2 therapies is the goal of future research and a limitation of this work. Finally, our analyses account for comorbidities with higher prevalence in patients with immunodeficiency compared with the general population (e.g., chronic lung disease); other comorbidities such as obesity, diabetes, ischemic heart disease, smoking status, and mental health status have been linked to the risk for long COVID in the general population and will be the focus of future research.

Nonetheless, findings from this study suggest the potential benefit of acute nirmatrelvir/ritonavir use in reducing the risk of long COVID among immunodeficient patients. Future prospective studies are essential to confirm nirmatrelvir/ritonavir’s protective effect against long COVID in immunodeficiency patients. Randomized controlled trials focusing on this high-risk population may clarify how nirmatrelvir/ritonavir influences long COVID development and determine the optimal timing and duration of antiviral therapy.

## Data Availability

The data used for this study are not publicly available due to an effort to protect patient privacy. The data may be available to share by the corresponding author upon request, only as additionally permitted by an Institutional Review Board–approved data use agreement.
